# Integrating a single-dose intravenous iron therapy (ferric carboxymaltose) for maternal anaemia in Nigeria: Insights from stakeholder engagement

**DOI:** 10.4102/jphia.v17i1.1672

**Published:** 2026-02-05

**Authors:** Rachel A. Thompson, Uchenna Gwacham-Anisiobi, Chisom Obi–Jeff, Mobolanle Balogun, Opeyemi R. Akinajo, Esther Oluwole, Yusuf Adelabu, Temitope Audu, Bosede B. Afolabi, Aduragbemi Banke–Thomas

**Affiliations:** 1 Centre for Clinical Trials, Research and Implementation Science, Lagos, Nigeria; 2 Brooks Insights Limited, Abuja, Nigeria; 3 Department of Community Health and Primary Care, Faculty of Clinical Sciences, University of Lagos, Lagos, Nigeria; 4 Department of Obstetrics and Gynecology, Faculty of Clinical Sciences, University of Lagos, Lagos, Nigeria; 5 Department of Medicine, Faculty of Clinical Sciences, University of Lagos, Lagos, Nigeria; 6 Department of Infectious Disease Epidemiology and International Health, Faculty of Epidemiology and Population Health, London School of Hygiene and Tropical Medicine, London, United Kingdom

**Keywords:** maternal anaemia, intravenous iron, stakeholder perspectives, pregnancy, service integration, Nigeria

## Abstract

**Background:**

Maternal anaemia contributes to high maternal morbidity and adverse birth outcomes in Nigeria. Oral iron supplementation is common but limited by poor adherence, side effects and systemic barriers. Ferric carboxymaltose (FCM), a single-dose intravenous iron formulation, offers a promising alternative, yet its routine use in Nigeria remains limited. Policymakers, providers and community leaders influence adoption of health services, making their perspectives key to successful integration.

**Aim:**

To explore stakeholder perspectives on integrating FCM for maternal anaemia into routine maternal health services.

**Setting:**

The study was conducted in Lagos State, Nigeria.

**Methods:**

A qualitative descriptive study was conducted. Thirty-three purposively selected stakeholders participated, representing government agencies, healthcare providers, academic institutions, non-governmental organisations, professional associations and community leaders. Participants were grouped by affiliation and roles to encourage open dialogue. Discussions followed a semi-structured guide, were audio-recorded, transcribed verbatim and thematically analysed using an inductive approach.

**Results:**

Three interrelated themes emerged: (1) health workers are not sufficiently prepared for intravenous iron delivery but could be through sensitisation, reorientation and resourcing; (2) sociocultural and religious influences, alongside misconceptions, require targeted community engagement and (3) affordability is a barrier but could be addressed through strategic policy levers.

**Conclusion:**

Successful integration of FCM requires investment in health system capacity, sustained community engagement and alignment with financing and policy frameworks.

**Contribution:**

The findings illustrate how stakeholder-informed analysis can reveal the practical, cultural and financial conditions necessary for sustainable uptake of FCM, advancing understanding of how maternal health innovations can be integrated within fragmented health systems.

## Introduction

Anaemia in pregnancy remains a significant public health concern in low- and middle-income countries (LMICs), including Nigeria, where prevalence ranges from 25% to 45%.^1,2^ In Nigeria, the burden of maternal anaemia is not evenly distributed, with evidence of higher prevalence in underserved urban slums and rural areas compared to more affluent communities,^3^ emphasising how structural inequities magnify the risks faced by already vulnerable women. Iron deficiency is the leading cause of anaemia in pregnancy and contributes substantially to maternal morbidity and mortality.^2^ Although oral iron supplementation is widely recommended, it is often undermined by poor absorption, gastrointestinal side effects and low adherence.^4^ Intravenous (IV) iron, especially ferric carboxymaltose (FCM), offers advantages such as rapid iron repletion, fewer side effects, higher tolerability and reduced dosing frequency.^4,5^ Emerging evidence suggests that intravenous FCM is more effective than oral ferrous sulphate in reducing iron deficiency and anaemia during pregnancy, with a comparable safety profile.^6^

Despite the existing evidence of clinical efficacy and safety, IV iron therapy, including FCM, is not routinely implemented in Nigeria. This integration gap, as in other LMICs, highlights a key challenge, as its adoption requires more than clinical justification. While several intravenous therapies are widely administered in Nigerian health facilities, IV iron presents distinct implementation issues, including evidenced risk of anaphylaxis with older formulations and perceived similarity to blood transfusion.^7^ Therefore, this necessitates an understanding of how the intervention aligns with local health systems, provider practices and sociocultural contexts.^8^ This is especially relevant in Nigeria, where decentralisation, system fragmentation and resource competition have constrained the effective delivery of healthcare, including maternal health innovations.^9^

Stakeholders influence policy, service delivery and community uptake, and their insights are crucial for tailoring implementation strategies to local contexts. As such, engaging stakeholders is vital to navigating this integration gap. In Nigeria, where antenatal care uptake varies^3^ and injectable therapies are sometimes mistrusted,^10^ stakeholder engagement can uncover context-specific factors that affect the integration of new interventions into routine care. While clinical trials have established the safety and efficacy of FCM,^6^ limited evidence exists on the perspectives of women, providers and policymakers who ultimately determine whether such interventions can be successfully integrated into existing health systems.^8,11^ This study explores stakeholder perspectives on integrating FCM for maternal anaemia into routine maternal health services in Lagos State, Nigeria. Insights from this study can offer guidance for service integration efforts that are both context sensitive and system aware, contributing to broader efforts to strengthen implementation strategies in similar health systems.

## Research methods and design

### Study design and setting

This study employed a qualitative descriptive design to explore stakeholder perspectives on the implementation of FCM for maternal anaemia.^12^ This approach was selected to gain an in-depth understanding of stakeholder views, practical considerations and the perceived barriers and facilitators to integrating FCM into routine maternal health services. This study formed part of the preparatory phase of the Implementation Research for Intravenous Iron Use in Nigeria (IVON-IS) project, which aims to test strategies for improving the prevention and treatment of anaemia during pregnancy and postpartum, including the routine administration of FCM.^13^ The study was guided by implementation research principles, which emphasise the involvement of key stakeholders in identifying context-specific strategies for introducing and sustaining health innovations.^14^

The IVON-IS project was conducted in Lagos State, southwestern Nigeria.^13^ Lagos State is Nigeria’s largest and most populous urban centre, with over 20 million residents, reflecting wide disparities in income, education, infrastructure and health access.^15^ In urban slum settings like Makoko, women’s use of maternal health services is often shaped by socioeconomic circumstances, including education, income, employment and proximity to health facilities.^16^ The prevalence of anaemia among pregnant women in Lagos is estimated at approximately 50%.^17^

Lagos state has a complex health system landscape, characterised by a three-tiered structure of primary, secondary and tertiary care delivered through both public and private sectors, alongside influential community-based providers. Governance is coordinated by the Ministry of Health and the Primary Health Care Board, with the State Health Management Agency working to extend financial protection and coverage through social health insurance schemes targeted at women and children. International and development partners contribute to strengthening reproductive, maternal, new born, child and adolescent health as well as primary health care through long-term programmatic support. Engaging across this diverse ecosystem is essential for tailoring integration strategies to different socioeconomic groups, ensuring feasibility and scalability across multiple levels of the system, aligning with institutional priorities and building trust with community actors who shape health-seeking practices.

### Participant selection and recruitment

Participants were purposively selected based on their relevance to maternal health service delivery, policy influence and community engagement. A Responsible-Accountable-Consulted-Informed (RACI) matrix was systematically developed through iterative consultations with different maternal health stakeholders to guide the identification of stakeholders likely to influence or be affected by FCM implementation.^14^ The matrix mapped stakeholders across four dimensions: those who would deliver IV iron therapy (Responsible), those with decision-making authority and approval power (Accountable), those whose input would be sought (Consulted) and those to be kept informed of the study progress (Informed). The application of the RACI matrix allowed us to go beyond broad stakeholder categories by clarifying which individuals were most likely to influence outcomes through direct delivery, decision-making authority or informal community influence, thereby ensuring a balanced representation of perspectives This systematic mapping identified 35 potential stakeholders, of which 33 were purposively selected to ensure representation across these different roles in the implementation process.

Stakeholders included policymakers, health service managers, healthcare providers, academic researchers, staff of non-governmental organisations (NGOs), professional association leaders and community representatives. They were purposively selected to capture diverse perspectives across decision-making, service delivery and community engagement.

Different groups were approached using tailored strategies: policymakers and managers via official channels and follow-up calls; healthcare providers through facility heads and professional associations; researchers and NGO staff via institutional networks and community representatives through local leaders and organisations. Invitation letters were accompanied by participant information sheets explaining the study’s purpose, procedures and confidentiality, as well as the voluntary nature of participation and the option to withdraw. Logistical support, such as transport stipends, was provided to facilitate attendance at the stakeholder workshop where data collection occurred.

### Data collection

Data collection took place in November 2022 through focus group discussions (FGDs) involving 33 participants, with each group comprising six to eight people. Group composition reflected different stakeholder categories. Researchers trained in qualitative methods (including Chisom Obi–Jeff, Mobolanle Balogun and Yusuf Adelabu) facilitated the discussions using a semi-structured guide that explored how IV iron therapy for maternal anaemia could be integrated into routine maternal health services in Lagos State, Nigeria. The discussion guide covered potential facilitators and barriers to integration, strategies for addressing these barriers and approaches for sustaining stakeholder engagement. All discussions were conducted in English, audio-recorded with participant consent, supported by notetakers and lasted between 60 min and 90 min.

### Data management and analysis

Audio recordings were transcribed verbatim and subjected to thematic analysis using an inductive approach.^18^ Two researchers (Mobolanle Balogun and Esther Oluwole) independently reviewed the data, developed initial codes and iteratively refined a shared codebook. Codes were grouped into categories and synthesised into broader themes. Data saturation was reached when additional data collection yielded no new information.^18^ Data analysis was supported by qualitative software (Dedoose Version 9.0.107, SocioCultural Research Consultants, LLC, Los Angeles, CA, United States). Analytical rigour was ensured through dual coding, peer debriefing and consensus meetings within the research team. In addition, regular reflective debriefings were held after each discussion to consider potential biases, compare impressions and refine interpretations, which supported the development of a shared analytic lens before formal coding commenced.

The positionality of the research team was also considered during the study. Several members of the team are maternal health researchers and clinicians with long-standing involvement in Nigeria’s health system, which not only provided valuable contextual understanding but also carried the risk of shaping assumptions about service delivery realities. Others had backgrounds in social science and implementation research, which helped to balance clinical perspectives with attention to sociocultural and systemic dynamics. To mitigate potential bias, the team engaged in reflexive discussions throughout the study, acknowledging their professional orientations and how these might influence data interpretation.

### Ethical considerations

Ethical clearance to conduct this study was obtained from Lagos University Teaching Hospital Health Research and Ethics Committee (No. ADM/DCST/HREC/APP/5328) and the Federal Medical Centre, Ebute Metta (No. HREC 22-22). The Lagos State Health Service Commission and the Lagos State Primary Health Care Board granted operational approval. Written informed consent was obtained from all participants following a detailed explanation of the study objectives, procedures and confidentiality processes.

## Results

Thirty-three participants from seven stakeholder groups participated in the study. These comprised 7 policymakers, 10 health service managers, 2 healthcare providers, 2 academic researchers, 3 NGO representatives, 3 representatives of associations or interest groups and 6 community representatives. Participants included both men and women across all groups.

Thematic analysis yielded three interrelated themes reflecting stakeholder perspectives on integrating FCM for maternal anaemia into routine maternal health services in Lagos State: (1) health workers are not sufficiently ready but can be with sensitisation, reorientation and equipping; (2) sociocultural, religious influences and misconceptions need to be met with community engagement and (3) affordability is of concern but can be mitigated by policy levers.

### Health workers are not sufficiently ready but can be with sensitisation, reorientation and equipping

Healthcare providers and health service managers acknowledged the clinical value of FCM, but expressed reservations about the system’s readiness to deliver it at scale. Concerns focused on gaps in human resources, infrastructure and logistics. Many stakeholders highlighted the need for sensitisation and training of health workers:

‘Sensitisation at the facility level, the healthcare team, apart from the ones that came for this meeting, should be sensitised.’ (Male, Healthcare provider, Secondary health facility)

‘Educating everyone that will be involved, including the patients, is essential.’ (Female, Academic researcher, tertiary institution)

Resistance to change among health workers, coupled with lingering fears about side effects, emerged as significant barriers to integration. Participants noted that even within clinical settings, entrenched misconceptions about the safety of IV iron more generally could undermine FCM uptake. Some healthcare workers were perceived as reluctant to adopt new practices, especially when such practices challenged existing routines or beliefs:

‘The attitude of the healthcare workers can be a barrier because we know that people resist change.’ (Female, Association/interest group, Professional body)

‘One thing that we need to address is the existing myths and beliefs among the healthcare workers and patients. It could be fear of side effects from the drug by the healthcare workers or the fear of injection by the patients.’ (Female, Academic researcher, tertiary institution)

Adequate infrastructure and reliable access to commodities were also raised as critical for delivery. Stakeholders stressed that successful integration would require equipping laboratories, ensuring consistent stock at facility level and maintaining the materials needed for safe administration:

‘Availability of logistics; availability of equipment and materials to ensure the things required will be available, and the lab should be equipped.’ (Female, Community representative, Ward Health Committee)

Beyond initial readiness, participants emphasised that ongoing education and feedback loops are essential to sustain confidence and ensure consistent delivery across facilities:

‘Regular feedback to each stakeholder, including facilities, will ensure continuous engagement.’ (Female, Academic researcher, tertiary institution)

### Sociocultural, religious influences and misconceptions need to be met with community engagement

Healthcare providers and health service managers consistently emphasised the importance of cultural and religious beliefs in shaping patient attitudes towards IV iron therapy, including FCM. Low literacy and fears related to injections, mistrust of medications administered intravenously and religious objections to blood-like substances emerged as barriers to uptake. In some communities, participants noted that the colour of the medication could trigger resistance unless proactively explained:

‘Even though in this instance, IV iron is not a barrier to some religions like Jehovah’s Witnesses, the colour of the intervention may be a barrier as it could be wrongly perceived as blood unless there is accurate sensitisation or education.’ (Male, Health service manager, tertiary facility)

Beyond specific misconceptions, fatalistic attitudes shaped by cultural norms were also identified as major obstacles to early antenatal engagement and adherence to FCM. Some participants further highlighted broader belief systems that discourage health-seeking altogether:

‘There is this culture and belief that you do not need to do anything to care for yourself. Some people believe that whatever comes to you, you should take it, and if you are going to die, you will die, with nothing to be done to prevent it.’ (Female, NGO representative, Community-based organisation)

Participants emphasised the critical role of trusted community actors in promoting awareness, generating sustained demand and facilitating acceptance of FCM by navigating local cultural sensitivities. Traditional birth attendants (TBAs), ward development committees (WDCs) and religious leaders were identified as essential connectors between the community and health facilities, leveraging their established relationships and deep understanding of community norms to support ongoing uptake and integration into routine care:

‘The TBAs in the community will be able to identify those who will benefit from the treatment and encourage them to go to the health facility.’ (Female, Health service manager, tertiary facility)

‘The WDCs are very instrumental in mobilising their communities. With a small incentive, they can take your work very far.’ (Female, Association/interest group, Professional body)

Community sensitisation was regarded as essential not only to raise awareness but also to generate grassroots ownership. Participants stressed that sustained engagement of communities, through recognition, modest financial support or small incentives, would be critical for maintaining trust and participation:

‘We need to find the stakeholders; the pregnant women should have a buy into the programme … we need to sensitise the community and community participation should be prioritised.’ (Male, Policy maker, State government)

‘There should be motivations for the mothers in the form of souvenirs or transport fare. This will also help with publicity.’ (Female, NGO representative, Community-based organisation)

### Affordability is of concern but can be mitigated by policy levers

Concerns about the affordability and cost-effectiveness of FCM emerged consistently across stakeholder groups. While FCM was provided free of charge during the study, stakeholders questioned whether patients could afford the treatment and whether facilities could absorb the procurement and delivery costs, noting that its high unit cost could pose a barrier to sustainability:

‘Cost of the IV iron treatment [*FCM*] matters. Will it require admission of the patient? Needs to be considered.’ (Female, Health service manager, Private health facility)

In response, several participants suggested leveraging existing health financing mechanisms, particularly the National Health Insurance Scheme (NHIS) and Lagos State health insurance schemes, to ensure access and equity:

‘The treatment could be listed on the National Health Insurance Scheme.’ (Male, Policy maker, State government)

Stakeholders also linked cost to the reliability of supply, noting that inconsistent procurement and distribution could exacerbate financial burdens on patients and undermine uptake:

‘Availability of IV iron [*FCM*] will ensure the project’s success and sustenance, and its unavailability will be a barrier at all levels.’ (Male, NGO representative, Community-based organisation)

As a structural policy response, several participants advocated for local pharmaceutical production of FCM. This was seen as a means to reduce importation costs, improve supply reliability and enhance affordability over time:

‘Local manufacturing of IV iron will reduce the cost of importing it from outside the country and improve its availability.’ (Female, Academic researcher, tertiary institution)

## Discussion

This study explored stakeholder perspectives on the integration of FCM into routine maternal health services, aiming to understand practical considerations, perceived barriers and potential facilitators. Findings from the FGDs highlighted several factors that could influence the feasibility of integration, including the readiness and capacity of the health system, provider knowledge and attitudes, sociocultural and community acceptability and financial and policy considerations.

The concerns of stakeholders in our study about the readiness of health facilities, staff capacity and supply chain reliability reflect a broader pattern observed in maternal health programmes across sub-Saharan Africa.^19^ Introducing an IV therapy into routine maternal care requires careful logistical coordination, sustained availability of commodities and alignment with existing provider workflows. Evidence from a Nigerian study highlighted that the consistent supply of FCM was a critical factor in achieving high intervention fidelity.^6^ Without these enabling conditions, even technically sound interventions often face limited uptake and inconsistent integration into routine services. Stakeholder perspectives on integrating FCM are possibly influenced by Nigeria’s history with maternal health interventions. Past challenges, including stock-outs, inconsistent implementation and poor integration of maternal health therapies, have fostered caution among providers and communities.^20^

While healthcare providers and health service managers stressed the importance of addressing infrastructural and supply chain gaps, they also noted that structural readiness alone could be insufficient. Even when facilities are adequately equipped and essential commodities are available, behavioural and institutional factors such as provider resistance, entrenched routines and a limited sense of ownership can undermine adoption. Healthcare worker reluctance, often shaped by uncertainty about new interventions and the comfort of established practices, reflects challenges observed during the roll-out of other maternal health interventions, including misoprostol and magnesium sulphate.^21,22^ Training by itself is unlikely to overcome these barriers in contexts where organisational culture discourages change. Implementation strategies must therefore include mechanisms for peer learning, clinical mentoring and role modelling that help shift provider norms over time and build confidence in new practices.^23^

In parallel, this study underscores that social meaning matters, and aligning innovations with local cultural logics is essential for uptake. Stakeholders emphasised that maternal health interventions cannot be abstracted from the sociocultural settings in which they are delivered. In this case, the symbolic features of FCM, such as its colour and the use of an intravenous drip, carry social meanings that can be misinterpreted as blood replacement, triggering religious or cultural objections. This symbolic interpretation of FCM’s colour represents a challenge unique to this specific IV iron therapy and is distinct from patient objections typically encountered with other clear IV solutions.^24^ Similar dynamics have been observed elsewhere, where interventions are rejected not because of their medical rationale but because of the meanings they evoke. Building on these sociocultural reflections, a key principle in implementation science becomes evident: acceptability is co-produced through relationships, not simply delivered through information.^25^ Similarly, community representatives and NGO representatives emphasised the role of community actors such as traditional birth attendants and religious leaders who possess the credibility and embeddedness to navigate local concerns. Their inclusion is not an outreach strategy but a recognition of distributed health authority in pluralistic health systems.^26^ For sustainable uptake, implementers must move beyond top-down sensitisation campaigns and instead foster co-creation processes that embed the intervention within community values and networks.

Participants in this study emphasised that financing and policy integration of FCM are critical determinants of equitable access. Systemic gaps in infrastructure, supply chains and governance reinforce expectations of logistical hurdles, which have been reported in the country, highlighting the need for comprehensive system strengthening to support successful integration.^27^ Cost, in particular, was identified as a major factor influencing both feasibility and equity, aligning with global evidence that user fees and out-of-pocket payments continue to pose significant barriers to maternal health services in LMICs.^28^ Although FCM was supplied at no cost during the study, participants questioned the sustainability of this model. They pointed to existing health financing schemes, including the NHIS, as potential vehicles for integration. However, as past evaluations of the NHIS have shown, mere inclusion in benefit packages does not guarantee access without concurrent attention to service readiness, provider reimbursement and enrolment equity.^29^ Discussions around financing also revealed tensions between short-term affordability and long-term sustainability, suggesting that while inclusion in health insurance packages could reduce immediate out-of-pocket costs, a parallel commitment to reliable supply chains and local production capacity would be necessary to maintain equitable access. The proposal for local manufacturing of FCM aligns with national pharmaceutical policy goals and offers a potential solution to price and supply volatility. Yet, this strategy requires substantial investment, regulatory oversight and coordination between the health and industrial sectors. As the literature on local production cautions, cost savings are not automatic and must be weighed against quality assurance and economies of scale.^30^

### Strengths and limitations

A key strength of this study is the diversity of stakeholder representation, which allowed for the integration of multiple perspectives spanning clinical, policy, community, non-governmental and administrative domains. This breadth of participation enriched the analysis and improved the relevance of the findings for real-world integration and implementation. The use of structured facilitation, dual coding, peer debriefing and consensus meetings during analysis enhanced the depth, credibility and dependability of the data.^18^ Although community representatives were included, women themselves, the primary beneficiaries of FCM, were not directly engaged, which may have limited the capture of their perspectives.

While the study benefited from engaging stakeholders before the start of integration activities, its reliance on a single engagement event limited the ability to capture evolving perspectives over time. The absence of follow-up interviews restricted opportunities to explore how stakeholder views might change during and after integration, thereby constraining the temporal depth of insights. Despite these limitations, the thematic consistency observed across groups and the integration of diverse voices contribute to the trustworthiness and transferability of the findings.

## Conclusion

Successful integration of FCM for maternal anaemia in Nigeria requires coordinated attention to system capacity, provider readiness, community trust and financial accessibility. These elements are not ancillary to implementation, but constitute its foundation. Policymakers, funders and implementers must therefore approach FCM not only as a therapeutic innovation but as a test of system’s responsiveness to maternal health needs. Addressing the systemic barriers, both generic health system issues and those peculiar to FCM, is essential. However, the study findings emphasise that policy solutions must target the specific structural challenges of FCM, such as high unit cost and local production capacity, to achieve sustainable integration.
